# Risks Posed by Intercrops and Weeds as Alternative Hosts to *Xanthomonas campestris* pv. *musacearum* in Banana Fields

**DOI:** 10.3389/fpls.2018.01471

**Published:** 2018-10-10

**Authors:** Walter Ocimati, Evans Were, Jeroen C. J. Groot, Pablo Tittonell, Gloria Valentine Nakato, Guy Blomme

**Affiliations:** ^1^Farming Systems Ecology Group, Plant Sciences, Wageningen University & Research, Wageningen, Netherlands; ^2^Bioversity International, Kampala, Uganda; ^3^International Institute of Tropical Agriculture, Kampala, Uganda; ^4^Institute of Agricultural Sciences in the Tropics (Hans-Ruthenberg-Institute) (490), University of Hohenheim, Stuttgart, Germany; ^5^Agroecology, Environment and Systems Group, Instituto de Investigaciones Forestales y Agropecuarias de Bariloche (IFAB), INTA-CONICET, San Carlos de Bariloche, Río Negro, Argentina; ^6^Groningen Institute of Evolutionary Life Sciences, Groningen University, Netherlands; ^7^Bioversity International, Addis Ababa, Ethiopia

**Keywords:** alternative host, banana, *Canna* spp., maize, millet, sorghum, *Xanthomonas campestris pv. musacearum*, Xanthomonas wilt

## Abstract

Alternative host plants are important in the survival and perpetuation of several crop pathogens and have been suspected to play a role in the survival of *Xanthomonas campestris* pv. *musacearum* (Xcm) and perpetuation of Xanthomonas wilt (XW) disease of banana and enset. This study determined the potential risk posed by two weeds (*Canna* spp. and wild sorghum) and common banana intercrops (maize, millet, sorghum, taro, and sugarcane) as alternative hosts to Xcm. The study employed screenhouse experiments, laboratory procedures and diagnosis of banana fields in XW-affected landscapes. Typical XW symptoms were only observed in artificially inoculated *Canna* sp., with an incidence of 96%. Leaf lesions characteristic of xanthomonads occurred on millet (50%) and sorghum (35%), though the plants recovered. No symptoms occurred in maize, sugarcane, taro or wild sorghum. However, Xcm was recovered from all these plant species, with higher recoveries in *Canna* sp. (47%), millet (27%), sugarcane (27%), and wild sorghum (25%). Only isolates recovered from *Canna* sp., millet, sorghum and wild sorghum caused disease in banana plantlets. The presence and incidence of XW on-farm was positively associated with the presence of susceptible ABB *Musa* genotypes and negatively with number of banana cultivars on farm and household access to training on XW management. Only 0.02% of field sampled *Canna* spp. plants had Xcm. Risk posed by *Canna* spp. on-farm could be limited to tool transmission as it has persistent floral bracts that prevent insect-mediated infections. Given the high susceptibility, perennial nature and propagation through rhizomes of *Canna* sp., it could pose a moderate-high risk, thus warranting some attention in the management of XW disease. Sugarcane could offer a low-moderate risk due to its perennial nature and propagation through rhizomes while risk from maize, millet, and sorghum was deemed zero-low due to their annual nature, wind-mediated mode of pollination and propagation through seed. Understanding the interactions of a crop pathogen with other plants is thus important when diversifying agroecosystems. The study findings also suggest other factors such as cultivar composition and management of the disease at farm and landscape level to be important in the perpetuation of XW disease.

## Introduction

Xanthomonas wilt (XW) disease of banana (*Musa* spp.) and enset (*Ensete ventricosum*) caused by the bacteria *Xanthomonas campestris* pv. *musacearum* (Xcm) has severely affected the production of banana and plantain in the east and central African (ECA) region. Host range studies have shown all the edible *Musa* spp. and enset cultivars in this region to be susceptible, though the level of susceptibility has been observed to vary with genotypes (Michael et al., 2006; Ssekiwoko et al., 2006b; Kebede and Gemmeda, 2017). Only *Musa balbisiana*, a wild *Musa* sp., has been reported to be resistant (Ssekiwoko et al., 2006b). The potential inoculum sources of Xcm have been reported to include infected plants, infected planting materials, infected plant residues, traded banana products (fruits and leaves) and contaminated soils and water (Eden-Green, 2004; Karamura et al., 2008; Nakato et al., 2014). Efforts to manage XW disease in ECA have mainly focused on the banana crop, yet banana grows in association with other crop and weed species. Weed fallows and some food and/or fodder crops such as common beans (*Phaseolus vulgaris*), cassava (*Manhot esculent*), maize (*Zea mays*), taro (*Colocasse* spp.), sweet potato (*Ipomea batatas*), sorghum (*Sorghum bicolor*), tobacco (*Nicotiana tabacum*), and Napier grass (*Pennisetum purpureum*) have also been recommended for breaking the cycle of XW (Mwangi et al., 2006). Some of the weeds and crops in association with banana could potentially influence the XW dynamics either through inhibiting spread and survival of the pathogen or supporting pathogen survival and perpetuation of the disease. Understanding the nature of interactions of plants in the survival of pathogens and disease dynamics are thus important. Field level crop diversification of agroecosystems has been reported as a promising strategy for suppressing pests and diseases (Letourneau et al., 2011; Boudreau, 2013; Poeydebat et al., 2017). Intercrops affect disease dynamics by altering wind, rain, and vector dispersal; modifying the microclimate (mainly temperature and moisture); altering host morphology and physiology; and directly inhibiting the pathogen (Boudreau, 2013). In contrast, other plants in an agroecosystem could exacerbate and perpetuate the diseases of certain crops, especially when acting as alternative host plants.

Alternative hosts have been reported to play a crucial role in the perpetuation of several diseases in different crop species. For example, the Indian tomato leaf curl virus was identified in 13 common weed species through symptoms and TAS-ELISA and was effectively transmitted by *Bemisia tabaci* from these weeds to tomato (Ramappa et al., 1998). Similarly, Ocimati et al. (2017) reported sorghum to be affected by *Pythium* spp. causing root rots in beans, thus exacerbating the bean root rot problem in southwestern Uganda. In banana, *R. syzygii* subsp. *celebesensis* strains that cause banana blood disease, a wilt of banana, is associated with some *Heliconia* species (Elphinstone, 2005; Blomme et al., 2017a). *Ralstonia solanacearum* that causes Moko/Bugtok wilt in banana has a wide host range (Belalcazar et al., 2004) and was also isolated from *Heliconia* species in the Coto valley virgin forests of southwest Costa Rica, leading to the suggestion that Moko could have originally been endemic in these rainforests (Sequeira and Averre, 1961). *R. solanacearum* strains causing Moko/Bugtok disease are associated with Solanaceous hosts thus compromising the efficiency of fallow periods in disease management. Therefore, the removal of weeds that are alternative hosts is recommended (Romo et al., 2012).

Pathogenic Xanthomonas species have also been reported in some crops such as maize (De Cleene, 2008), sugarcane (*Saccharum* spp.) (Destefano et al., 2003), sorghum (Reddy, 2012), common beans (Mkandawire et al., 2004; Todorović et al., 2008) and sweet potato (Hernandez and Trujillo, 1990), all of which are commonly grown in the banana-based systems of ECA. Xcm has also been shown to be phylogenetically similar to *Xanthomonas vasicola* pv. *vasculorum* (Xvv) that is pathogenic to sorghum, maize and sugarcane (Aritua et al., 2008; Lang et al., 2017).

A number of studies have been conducted to understand how other plant species (weeds and cultivated crops) in the environment of banana and enset interact with Xcm (e.g., Yirgou and Bradbury, 1974; Ashagari, 1985; Ssekiwoko et al., 2006a; Aritua et al., 2008; Karamura et al., 2015; Chala et al., 2016). Whereas studies consistently report *Canna* spp., a common weed, to develop XW characteristic symptoms similar to those in banana after inoculation with Xcm (Ssekiwoko et al., 2006a; Chala et al., 2016), they give a mixed and less clear picture regarding the interaction of Xcm with the cereals, especially with maize.

Yirgou and Bradbury (1974), Ashagari (1985), and Ssekiwoko et al. (2006a) observed no symptoms in cereals after inoculation with Xcm isolates from enset and banana. In contrast, Aritua et al. (2008) reported a hypersensitive response (pathogenic reaction) at the inoculation points of maize while Karamura et al. (2015) reported characteristic XW symptoms in sugarcane but not in maize. These two studies were able to isolate Xcm from the maize plants 5 weeks after the inoculations. Rutikanga et al. (2016) reported the isolation of Xcm from maize, beans and sweet potato plant parts and soils around these crops, and mixed weed fallow. The most common weed species in the Rutikanga et al. (2016) weed fallow sites included *Bidens pilosa* L., *Tithonia diversifolia* (*Hemsl.*) A Gray, *Bothriocline ugandensis* (S. Moore) M.G. Gilbert, *Leonotis nepetifolia* (L.) R Br, *Coleus/Plectranthus kilimandschari* Gurke ex Engl, *Ricinus communis* (L.), *Crassocephalum vitellium* Benth, *Canna indica* (L.), *Galinsoga ciliate* (Raf.) Blake, *Commelina diffusa* Burm. F., and *Crassocephalum montuosum* (S. Moore) Milne-Redh. Though the Rutikanga et al. (2016) isolates were confirmed to be Xcm with PCR, they did not cause disease when inoculated into tissue cultured banana plantlets.

A more recent study by Chala et al. (2016) using three Xcm isolates obtained from cultivated enset, wild enset and banana reported typical disease symptoms 2–3 weeks after the inoculations with incidences of 40–67% in maize, 25–50% in sorghum, 13% in wild sorghum and 1–17% in millet (*Eleusine coracana*). However, some of the above studies did not report re-isolation of Xcm from these alternative host plants while Koch’s postulates were not reported in all the studies. Yet some plant species can act as symptomless carriers or non-hosts of pathogens (Katan, 1971; Schaad and Dianese, 1981; Gitaitis et al., 1998; Fassihiani, 2000). For example, naturally occurring weeds including *Amaranthus* sp., *Chenopodium album*, and *aubergines* were colonized to various degrees and determined as symptomless carriers of *Fusarium oxysporum* f. sp. *Lycopersici* that is pathogenic to tomato (Fassihiani, 2000). In addition to clarifying the above observations, the risk posed by these crop species under on-farm situations also needs to be examined.

The current study built on to the above studies by (i) determining the potential risk of selected weeds (*Canna* spp. and wild sorghum) and common banana intercrops [maize, millet, sorghum, sugarcane, and taro (*Colocasia esculenta*)] to harbor and/or succumb to Xcm in controlled experiments; (ii) the potential of Xcm isolates from these putative alternative hosts to re-infect banana plants; and (iii) the potential importance of the putative alternative hosts in the perpetuation of XW in banana in farmers’ fields. Synthesis of these findings will be helpful in informing the management of XW disease on farms in the ECA region.

## Materials and Methods

This study was conducted through laboratory, screenhouse and field studies. Screenhouse and laboratory studies were conducted at the National Agricultural Research Laboratories (NARL) located at Kawanda in central Uganda in 2015/2016. The screen house and laboratory studies were complemented through farm diagnostic studies in central Uganda, a hot spot for XW disease.

### Screenhouse Studies

A total of eight plant species that included five common banana intercrops (maize, millet, sorghum, sugarcane, and taro) and two weeds [*Canna* sp. and wild sorghum (*Sorghum versicolor*)] previously reported or suspected to harbor or succumb to Xcm infection under controlled screenhouse conditions were used in this experiment. Two months old east African highland (EAHB) banana cv. ‘Musakala’ (AAA genome) plantlets were used as the positive control. The choice of the banana cultivar to use was based on availability of tissue culture plantlets, as all banana cultivars in the region are susceptible to XW disease following infection by the Xcm pathogen. For each species, 60 plants were raised from either seed (maize, millet, and sorghum); rhizome/corm bits (taro), cuttings (sugarcane), rhizome (*Canna* spp.), small plantlets (wild sorghum), or tissue culture plantlets (banana). To rule out any latent Xcm-infection in vegetatively propagated plants, cross sections from the stems and or leaves were sampled, total DNA extracted as described by Mahuku (2004) and checked with PCR using Xcm GspDm-specific primers (Adriko et al., 2012). The plants were then grown in small pots (3 L in size) filled 3/4 full with pre-sterilized forest top soil mixed with sand in a ratio of 2:1 over a period of 1–2 months (depending on the crop species) before treatment application. Sand was added to improve drainage and aeration while the plants were regularly watered to provide adequate moisture for growth.

#### Inoculum Preparation

Xcm for the screenhouse study was isolated from a fresh sample of a banana pseudostem obtained from a plant that had only recently developed XW symptoms (not older than 2 days) in an infested field at NARL, Kawanda. One gram of the sample was aseptically cut off from the middle and inner portion of the pseudostem tissue and macerated with a mortar and pestle in 3 mL of sterile distilled water, serially diluted fourfold and 10 μL of each dilution plated on Yeast Peptone Glucose Agar (YPGA, Mwangi et al., 2007) media in Petri plates. Plates were sealed and incubated at 28°C for a period of 72 h. Single colonies with Xcm–characteristics (yellow, mucoid, and dome shaped) were carefully picked, streaked on fresh media and incubated as above. Resultant colonies were confirmed using Xcm-specific primers (Adriko et al., 2012) using PCR, and a suspension of the bacteria adjusted to 0.5 OD_600_ (∼1 × 10^8^ colony forming units) using a NanoDrop spectrophotometer (Thermo Fisher Scientific Inc., Pittsburgh, PA, United States) for the inoculation of plants.

#### Inoculation of Plants

Thirty maize, sorghum, millet, bean, *Canna* spp., and wild sorghum plants were inoculated after 1 month from emergence or potting while sugarcane, taro and banana were inoculated at 2 months after establishment An equal number of plants served as un-inoculated controls. Inoculations were done by injecting 100 μL of fresh Xcm inoculum using a sub-dermal syringe into the stem tissues at 15–20 cm height. Twelve inoculated plants per species were routinely observed for symptoms typical of XW whereas the remaining 18 plants were routinely sampled for laboratory analysis. An equal number of un-inoculated plants were, respectively, kept for observation and sampling. The screenhouse plants were observed for a 60 days period corresponding to the life span of the annual crops and covering adequately the time period in which XW symptoms in banana plantlets are manifested. Data collected included symptom characteristics, time from inoculation to symptom expression (i.e., incubation period) and symptom incidence. Mean incubation was computed as sum of XW incubation period for the individual symptomatic plants divided by the total number of symptomatic plants, while incidence was determined as the percentage of plants that showed XW symptoms over the study period.

#### Replications

The experiment was repeated thrice over the period of the study. Isolates for inoculation of plants in the three screenhouse experiments were obtained from the same field and thus assumed to be homogenous. The Xcm isolates for the first screenhouse experiment could not be used in subsequent experiments due to a possible change in their virulence associated with repeated culturing and long storage in the laboratory (Tripathi Leena, 2017, personal communication).

#### Sampling of Plants

Three inoculated plants per species in the screenhouse were sampled at an interval of 7 days starting at 14 days and ending at 49 days post inoculation for Xcm isolation in the laboratory. Samples were destructively collected in an aseptic manner by sterilizing knives and gloves with a solution of 15% (v/v) sodium hypochloride (NaOCl) between samples to prevent cross contamination. Further precaution was taken to sample the un-inoculated controls first, followed by the potential alternative host species and lastly the already known/susceptible Xcm host (i.e., banana). For each plant species, samples were obtained from the stems/leaves and below ground parts. Samples were stored separately in labeled plastic bags and transferred to the laboratory where they were processed immediately or stored at 4°C for later isolation.

### Laboratory Studies

#### Isolation of Xcm

Samples from the field were separately washed in running water, surface sterilized using 15% v/v NaOCl to eliminate any epiphytes and external Xcm contamination, rinsed with distilled water to remove excess NaOCl and blotted dry using paper towels. Approximately 3 g of each plant part/sample were cut and homogenized with a sterile mortar and pestle in 3 mL of sterile distilled water. 1 mL of this homogenate was serially diluted to 10^-3^, from which a 20 μL aliquot was spread plated on triplicate Petri plates of YPGA- containing antibiotics 5-fluorouracil and cephalexin (Mwangi et al., 2007). Plates were sealed, incubated at 28°C for 3 days and scored for presence or absence of colonies with Xcm characteristics. All Xcm-like colonies were streaked on fresh YPGA media and incubated as above to obtain pure cultures and confirmed through PCR using Xcm GspDm-specific primers (Adriko et al., 2012).

#### Genomic DNA Extraction From Xcm and Polymerase Chain Reaction (PCR)

Genomic DNA was extracted from Xcm-like colonies as described by Mahuku (2004). The integrity (concentration and purity) of DNA samples was determined using the NanoDrop 2000C spectrophotometer (Thermo Fisher Scientific Inc., Pittsburgh, PA, United States) and adjusted to 50 ng/μL, for PCR. The gDNA extracted from Xcm-like colonies was used as template in a PCR reaction using 265 bp GspDm-specific Xcm primers (Adriko et al., 2012). Amplification reactions were carried out in a 20 μL reaction volume with a final concentration of 0.3 μM of each of the forward and reverse primers, 1.5 mM MgCl_2_, 0.2 μM of each dNTPs (Promega, Madison WI, United States), 1× PCR green buffer, 1 unit of HotStarTaq Plus DNA Polymerase (Qiagen, Canada) and 2 μL of genomic DNA (50 ng/μL). The PCR amplification reactions were performed in the Eppendorf Mastercycler (Eppendorf AG, Hamburg, Germany) using the following program: an initial denaturation at 95°C for 3 min; 35 cycles consisting of 92°C for 20 s, annealing at 64°C for 15 s, extension at 72°C for 15 s; and a single final extension at 72°C for 3 min before cooling and holding at 4°C. Amplified PCR products were separated by electrophoresis in 1.5% w/v agarose gel in 1× TAE buffer at 150 V for 45 min. The gel was stained with ethidium bromide (0.5 μg mL^-1^) and the image captured using the GBOX Syngene gel documentation system (SYNGENE, UK). Samples with a 265-bp amplicon were selected and preserved on 2 mm glass beads in 80% v/v glycerol at -80°C for further studies.

#### Koch Postulate Trials

Koch’s postulate trials were conducted to determine if the Xcm-like bacterial isolates recovered from the potential alternative host plants inoculated with Xcm could cause disease in the original host (banana plantlets). Koch’s postulates are a stringent criterion that provides a framework for thinking about the proof of microbial disease causation and are widely used in plant pathology (Liu et al., 2016). The key elements include a specific association of the microbe with the disease state, scientific consistence of microbiological and pathological evidence, isolation of the microbe on culture media, and reproduction of disease following inoculation of the cultured organism into a host. To fulfill Koch’s postulate, Xcm-characteristic colonies re-isolated from the alternative host plants in the study that were confirmed positive for Xcm with PCR were sub-cultured and inoculated into 2 months old EAHB banana (cv. ‘Musakala’) plantlets using the procedures described above. Five banana plantlets of the same age, each having a total of four leaves were inoculated per Xcm inoculum source/isolate. Five banana plantlets inoculated with Xcm isolated from banana plantlets served as controls. All inoculated banana plantlets were regularly monitored for disease symptoms, time to symptom expression and symptom incidence as in the section above. XW severity at a scale of 0–1, 0 being no disease symptom and 1 being the highest severity score, was also assessed for some of the Xcm isolates used for the Koch postulate trials. The severity S for a given isolate was assessed as below.

(1) $$S=\frac{sP_{1}+sP_{2}+----+sP_{n}}{Np}$$

(2) $$sP=\frac{Li}{LN}$$

Where: S = severity score for a given Xcm isolate inoculated into a total of “Np” plants; sP = XW severity score for a single plant, with the number of plants inoculated with a single isolate varying from “1” to “n” plants; Np = total number of plants inoculated with a given Xcm isolate; Li = number of symptomatic leaves at the time of data recording; and LN = the total number of leaves per plant at inoculation.

### Assessment of the Field Risk/Relative Importance of the Potential Alternative Host Plants of *Xanthomonas campestris* pv. *musacearum* on Farm

The potential risk and relative importance of crops or weeds that either showed XW symptoms in the controlled experiments or from which active Xcm could be isolated were assessed on-farm through diagnosis of 63 randomly selected banana farms in XW endemic districts (Mukono, Wakiso, Kayunga, and Luwero) in central Uganda and expert knowledge. On farms, data was collected on: XW presence and incidence; presence of *Canna* spp. and wild sorghum in banana fields; and presence of common banana intercrops (maize, millet, sorghum, sugarcane, and taro) in banana fields and or farm. XW incidence on each farm was scored on mat (i.e., an underground banana rhizome from which one or more shoots emerge) basis at a scale of 0 to 100%. Data was also collected on other potential factors that could influence XW presence and incidence, and these included: presence of banana cultivars with ABB genome, the diversity of banana cultivars and presence of agroforestry trees. Banana cultivars with ABB genome are highily susceptible to insect-mediated XW infections (Tripathi and Tripathi, 2009; Blomme et al., 2017a) whereas, a high diversity of banana cultivars and presence of agroforestry trees were anticipated to cause a dilution effect and reduce insect vector access to the susceptible ABB banana types. XW management practices also play a crucial role in influencing disease presence and incidence on farm. Thus information on management of banana fields (e.g., weeding, removal of male buds, and removal of excess suckers), the history of XW, household access to training on XW management, and key XW cultural control practices applied on farm were also collected using farmer interview schedules. The on-farm studies sought to determine if a cause-effect relationship existed between the presence of the potential alternative host plant(s) of interest as an independent variable and (i) the presence/absence of XW and (ii) the incidence of XW on banana farms as response variables. Other possible explanatory variables to the above two response variables assessed on-farm included the time of exposure to XW, key XW management practices [male bud removal, single diseased stem removal (SDSR), and complete banana mat removal (CMU)], number of banana cultivars on farm/field, presence/absence of ABB banana types, presence of agroforestry trees, banana intercropping, access to information on XW and farmer/household access to training on XW management. During field diagnosis, *Canna* spp. plants were aseptically sampled for laboratory isolation and identification of Xcm as described in sections above. Priority was given to sample *Canna* spp. plants that exhibited suspicious Xanthomonas characteristic wilting symptoms.

Expert knowledge from five scientists with at least 7 years experience on XW epidemiology and management was used to develop XW risk scores varying between 0 (no risk) to 5 (high risk) for the different potential alternative host crops. In addition to the results of the experiments in this study, key plant species characteristics used for assessing the risk from the alternative host plants included their mode of pollination, persistence or non-persistence of floral bracts and male neuter flowers, potential for tool mediated Xcm spread, mode of reproduction and life span (annual vs. perennial). XW is also spread by insects that visit for pollen and nectar, thus plant species pollinated by insects were deemed a risk to XW spread while banana plants with persistent neuter flower and floral bracts have been found to escape the disease. Plants that reproduce through rhizomes can potentially pass the bacteria or disease to subsequent generations while susceptible perennial plant species are likely to offer a higher risk to the banana crop than the annual crops.

### Statistical Analysis

Data were compiled in MS Excel. The GenStat v. 12 statistical software (VSN International Ltd., 2009) was then used to obtain the analysis of variance ANOVA and to separate means [at 5% Least Significant Difference (LSD)] of the laboratory and screenhouse data. The R-Statistical package (R Core Team, 2013) was used to separately conduct a regression analysis between the response variables and the explanatory variables. A general logistic regression model was used to explore the relationship between the presence/absence of XW on-farm with the above explanatory variables, except the XW management practices and exposure time. XW management practices (SDSR and CMU) were not used as explanatory variables because they can either be introduced on-farm in response to XW presence or influence XW presence. A linear model was used to explore the relationship between XW incidences with the explanatory variables. The explanatory variables for each response variable were reduced to a few that significantly influenced the observed response through a backward stepwise regression process, each step dropping explanatory variables that contributed least to the observed response.

## Results

### Screenhouse and Laboratory Studies

#### Occurrence of Xanthomonas Wilt Symptoms

**FIGURE 1:**
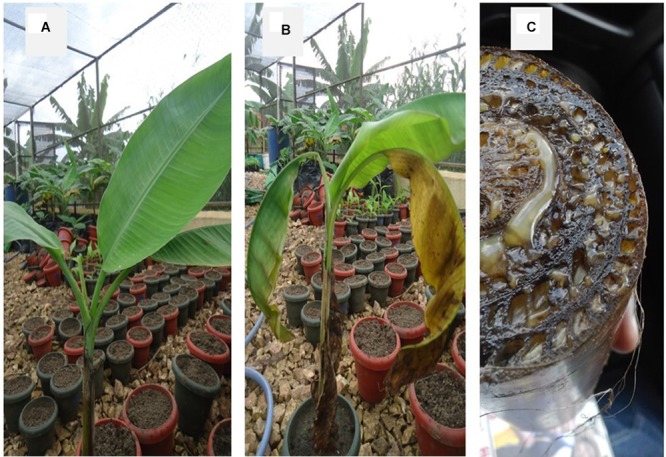
**(A)** A disease-free banana plant, **(B)** a banana plant showing Xanthomonas wilt symptoms after inoculation with *Xanthomonas campestris* pv. *musacearum*, and **(C)** a cut pseudostem of a XW infected banana plant showing yellow Xcm ooze.

**FIGURE 2:**
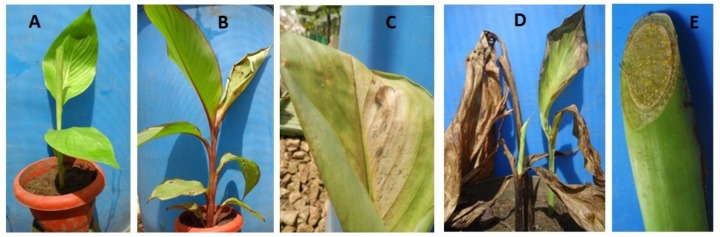
Photos of *Canna* sp. showing: **(A)** an un-inoculated control plant, **(B)** an inoculated symptomatic plant, **(C)** a symptomatic leaf, **(D)** a severely affected mat showing the dead parent plant (middle) and symptomatic shoots (foreground and behind), and **(E)** yellow bacterial ooze on a cut stem surface.

Characteristic symptoms similar to those of XW disease in banana (**Figure 1B**), i.e., progressive yellowing and wilting of leaves were only visible in *Canna* sp. plants (**Figures 2B,C**). In most of the cases, leaf necrosis and yellowing in *Canna* sp. started either in the middle part or edge of a leaf, subsequently progressing to the whole leaf and plant. Severely affected *Canna* sp. plants also had their leaf margins turning black, progressing rapidly to the entire leaf and plant (**Figure 2D**). The affected *Canna* sp. plants collapsed and rotted. Cut symptomatic *Canna* sp. pseudostems released a yellow bacterial ooze (**Figure 2E**) similar to cut XW infected banana stems (**Figure 1C**). Infections were also observed in some of the attached suckers following the inoculation of the parent *Canna* sp. plants (see **Figure 2D**) whereas other suckers from inoculated *Canna* sp. mats did not show symptoms. None of these symptoms occurred in the uninoculated banana (**Figure 1A**) and *Canna* sp. (**Figure 2A**) control plants.

**FIGURE 3:**
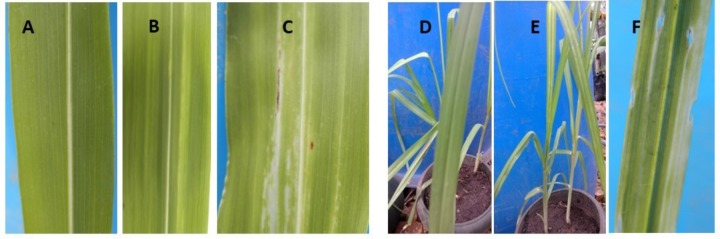
**(A)** The leaf of an un-inoculated control sorghum plant, **(B,C)** inoculated sorghum leaves with whitish lesions, **(D)** leaf of an un-inoculated millet plant, and **(E,F)** leaves of inoculated millet plants with white lesions. Photos depict plants/leaves at about 12 days after trial initiation/inoculation.

Leaf necrosis (whitish to light green lesions) along the parallel veins were observed on leaves of inoculated sorghum (**Figures 3B,C**) and millet (**Figures 3E,F**) plants, 7 days after inoculation. No symptoms occurred in the control plants (**Figures 3A,D**). However, these symptoms were observed to disappear over time and new emerging millet and sorghum leaves presented no leaf symptoms, suggesting that the plants recovered from the Xcm infection. No Xanthomonas-characteristic symptoms were observed in the controls or inoculated plants of maize, sugarcane, wild sorghum, beans or taro.

#### XW Incubation Period, Incidence, and Occurrence of Xcm in Plant Tissues

**FIGURE 4:**
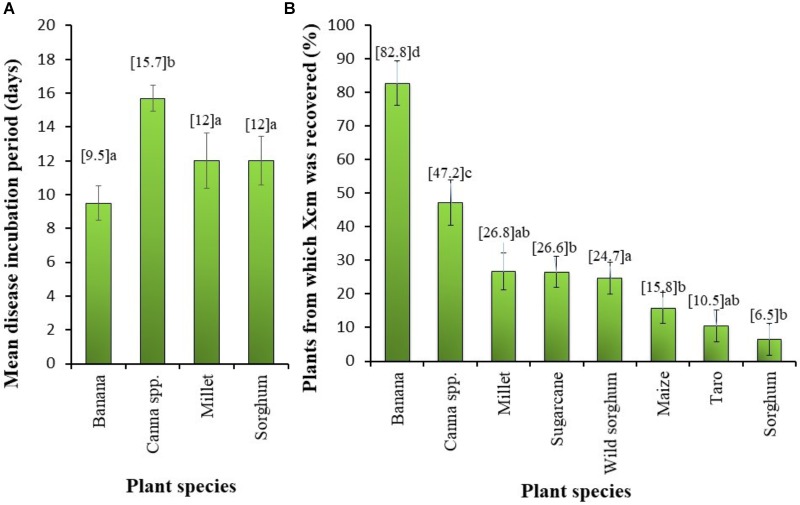
**(A)** Mean incubation period (i.e., time for the appearance of symptoms characteristic of Xanthomonas wilt) in different plant species and **(B)** the proportion of plants from different plant species from which *Xanthomonas campestris* pv. *musacearum* (Xcm) was recovered following a deliberate artificial inoculation. Vertical bars are standard errors. Significant differences were observed between the species means at *P* < 0.001. Mean values followed by the same letter are not significantly different at *P* = 5%.

The mean incubation period (number of days from time of inoculation to first symptom observation) was greatest for *Canna* sp. and did not differ significantly between banana, millet and sorghum (**Figure 4**). The XW symptom incidence in *Canna* sp. was 96%, while banana had 100% of potted plants showing symptoms. Millet had symptom incidence of 50% and sorghum 35%.

**FIGURE 5:**
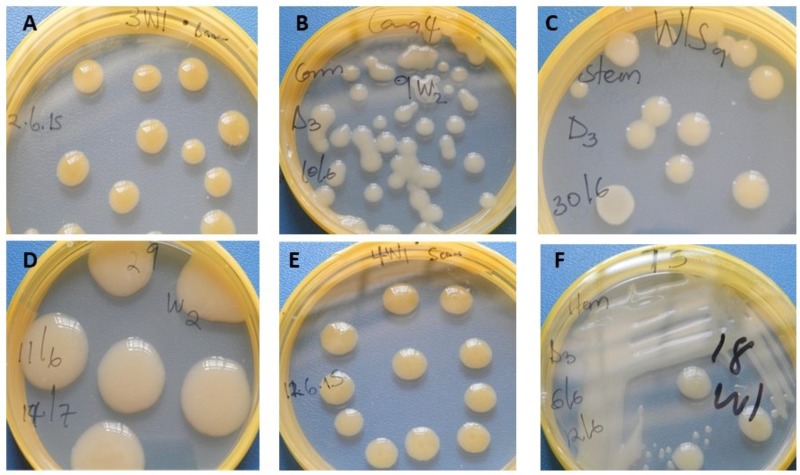
*Xanthomonas campestris* pv. *musacearum* (Xcm) like colonies (on Yeast Peptone Glucose Agar) recovered from different crop species artificially inoculated with Xcm. **(A)**, banana; **(B)**, *Canna* sp.; **(C)**, wild sorghum; **(D)**, sorghum; **(E)**, sugarcane; and **(F)**, taro.

**FIGURE 6:**
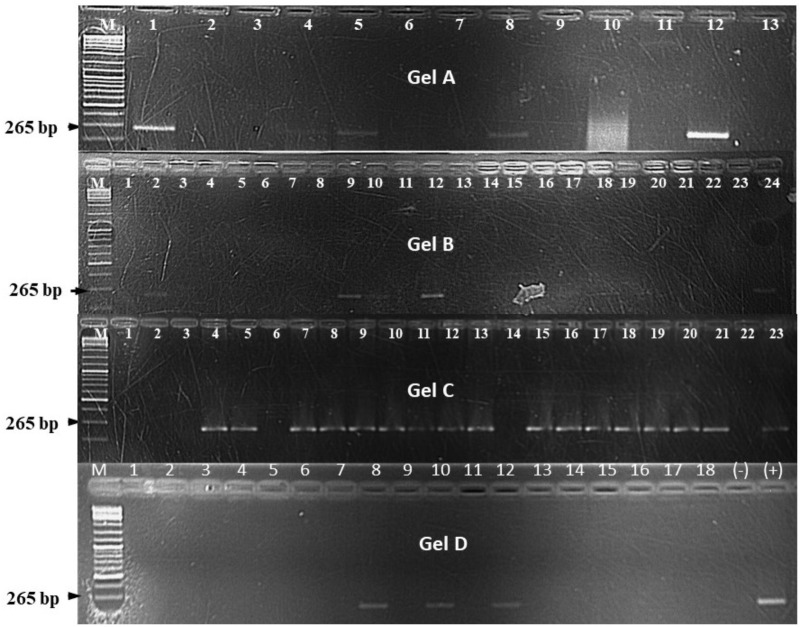
DNA bands on agarose gel for different *Xanthomonas campestris* pv. *musacearum* (Xcm) like isolates from different plant species after being inoculated with Xcm isolated from banana. On **gel A**, 1, *Canna* sp.; 2, 3, and 8, banana; 4, 5, 10, and 11, sugarcane; 6 and 7, taro; 9, maize; 12, positive control; and 13, negative control. On **gel B**, 1, 2, 7–9, and 13, wild sorghum; 3, 4, 5, and 16, maize; 6, 10, 12, and 17–21, *Canna* sp.; 11 and 14, sorghum; 15, sugarcane; 22, taro; 23, negative control; 24, positive control. **Gel C**, 1 and 2, sugarcane; 3, 5–7, 18, and 21, wild sorghum; 4, 16, and 20, taro; 8–10, 12, 13, 15, 17, and 19, banana; 11, *Canna* sp.; 14, sorghum; 22, negative control; and 23, positive control. On **gel D**, 1–18, millet. “M” on all the gels denotes the DNA ladder. The experiments were conducted at the Kawanda laboratory in central Uganda.

Xcm-like colonies (**Figure 5**) were re-isolated from both symptomatic and symptomless *Canna* sp., sorghum and millet plants and confirmed to be positive with a PCR using Xcm GspDm-specific primers (**Figure 6**; Adriko et al., 2012). Despite the absence of symptoms Xcm was also recovered from wild sorghum, sugarcane, maize and taro and similarly confirmed with the GspDm-specific primers (**Figures 5, 6**). Adriko et al. (2012) reported the GspDm-specific primers to be specific to Xcm based on a 265 bp amplicon. To our knowledge there are no other published primers other than GspDm reported to be specific for molecular diagnosis of Xcm. However, when we performed an *in silico* analysis with Primer-BLAST^1^, GspDm primers generated a specific amplicon of 265 bp with Xvv and non-specific amplicons (>3000 bp) with other Xanthomands (non-pathogens of banana). This suggests that the GspDm primers may also amplify Xvv that causes bacterial leaf streak in maize, sugarcane and sorghum. The fact that a 265 bp amplicon was not observed in the genomic DNA of tissues from maize, sugarcane and other plant species used in this study prior to inoculation gives us confidence that the plants used for the screenhouse experiments were Xvv free and amplicons obtained with Gspdm primers were Xcm.

The frequency of Xcm presence in the plant parts significantly (*P* < 0.001) varied between the crop species (**Figure 4B**). Xcm was recovered in 83% of the banana plant samples compared with 47% in *Canna* sp. plants. Xcm incidence in plant parts of the other crops was significantly lower than that in *Canna* sp. and varied between 6.5% in sorghum to 27% in millet (**Figure 4B**). For the period of Xcm isolation (13–39 days after inoculation), no consistent relationship was observed between the proportion of plants from which Xcm was recovered and the age of plants or time of Xcm re-isolation.

### Koch’s Postulates Using Pathogens Re-isolated From Non-host Plants

**FIGURE 7:**
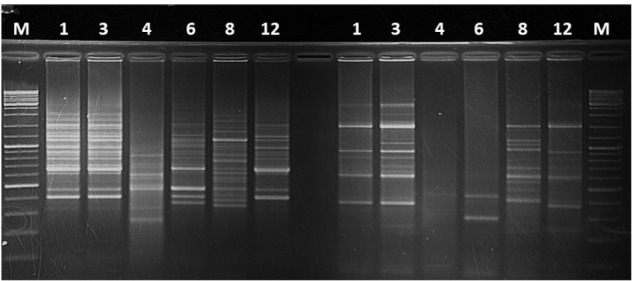
DNA finger prints on agarose gel for different *Xanthomonas campestris* pv. *musacearum* (Xcm)-like isolates from: 1, *Canna* sp.; 3 and 8, banana; 4, sugarcane; 6, Taro; and 12, Xcm positive control. The initial Xcm isolate was obtained from a symptomatic banana plant at Kawanda research station, central Uganda. Isolates 1, 3, and 8 caused disease in banana plantlets while 4 and 6 did not.

Koch’s postulates were performed using the isolates recovered from the different artificially inoculated crop species. Isolates recovered from banana, *Canna* sp., millet, wild sorghum and sugarcane caused XW symptoms in banana plantlets with incidences of 100% for those from banana, 60% for those from *Canna* sp., 80% for those from millet, 40–100% for those from wild sorghum and 40% for those from sugarcane. Isolates from maize and taro did not cause disease in banana while isolates from sorghum were not introduced into the banana plants. Finger-prints were obtained for some of the isolates from banana, sugarcane, *Canna* sp. and taro. Similar finger-print patterns were observed for the banana and *Canna* sp. isolates that caused disease in banana plantlets, while a different pattern was observed for the taro and sugarcane isolates that did not cause disease in banana plantlets (**Figure 7**).

**FIGURE 8:**
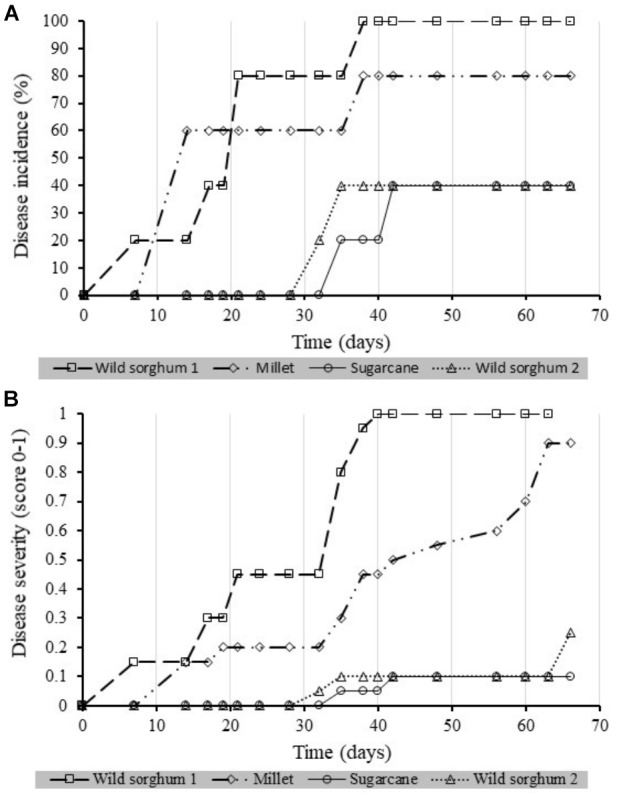
Xanthomonas wilt incidence [**(A)** %] and **(B)** severity scores over time in banana plantlets inoculated with *Xanthomonas campestris* pv. *musacearum* re-isolated from different Xcm non-host plant species. A total of five banana plantlets were inoculated per isolate. Severity was scored at a scale of 0 to 1, 0 being no disease and 1 being the highest severity scores.

Four of the Xcm isolates recovered from the potential alternative hosts were compared for their virulence on banana plantlets (**Figures 8A,B**). One isolate from wild sorghum was highly virulent causing up to 100% incidence with a correspondingly high severity score of 1 (1 being the highest score and 0 being no disease). A second isolate from wild sorghum and the isolate from sugarcane were the least virulent while the isolate from millet was moderately virulent.

### Xanthomonas Wilt Field Risk Drivers

**FIGURE 9:**
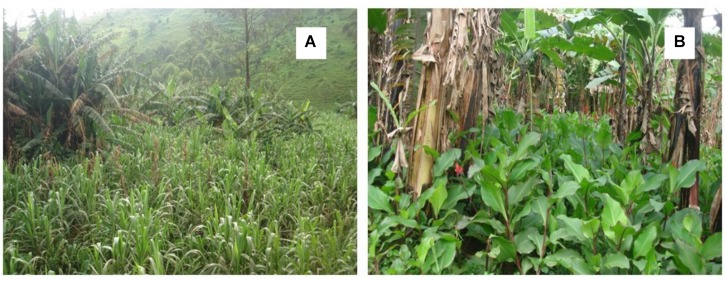
**(A)** Banana being intercropped with sugarcane, **(B)** a banana field with a high density of *Canna* sp.

Xanthomonas wilt was present in 92% of the surveyed farms with a mean mat disease incidence and time of exposure to XW of 21% and 4.7 years, respectively. The number of *Musa* cultivars on farm varied between 1 and 11, while the ABB types were present on 95% of the banana farms (**Table 1**). Agroforestry trees and intercropping were practiced on all the surveyed farms. Between 32 and 84% of the farmers practiced different XW control measures while between 5 and 76% of the farmers reported to have accessed information and or training from different sources. Farmers were the main source of information (75%) on XW disease (**Table 1**). *Canna* spp. was observed on 59.5% of the farms surveyed in central Uganda (**Table 1**). The abundance of *Canna* spp. on farms could not always be easily ascertained as most farmers had cleaned their fields using mainly hand hoes and/or herbicides in a few cases. In some farms (see example of **Figure 9B**), *Canna* spp. are very abundant. Out of 46 *Canna* spp. samples analyzed in the laboratory, only one plant (∼0.02%) had colonies characteristic of Xcm that were also confirmed positive using Xcm specific primers. The other samples either had no Xcm-like colonies or were negative on PCR with the specific primers.

**Table 1:** Distribution of response and explanatory variables explored on Xanthomonas wilt (XW) infected farms in central Uganda.

Variable	Mean value
XW presence on farms (%)	91.9
XW mat incidence score (%)	21.3 (0–80)
Time of exposure to XW disease (years)	4.7 (0–20)
Area under banana (hectares)	0.61 (0.10–4.05)
Number of banana cultivars on farm	4.4 (1–11)
Farms with ABB types of banana (%)	94.6
Presence of agroforestry trees (%)	100
Farms intercropping banana (%)	100
Farms applying complete mat uprooting (CMU) (%)	37.8
Farms applying single diseased stem removal (SDSR) (%)	32.4
Farmer accessing information on XW from other farmers (%)	75.7
Farmer accessing information on XW from extension agents (%)	5.4
Farmer accessing information on XW from media (%)	46.0
Households accessing training (%)	32.4
Farms on which *Canna* sp. was present (%)	59.5
Who accesses information in household (%)	
*- Men*	48
*- Women*	41.7
*- Children*	10.4
Male headed households (%)	83.8
Commercially oriented farms (%)	13.5


*In parenthesis are minimum and maximum values of the variables. A total of 63 farms were surveyed.*

A logistic regression model of XW presence/absence on farm only selected farmers access to training and the presence of ABB banana cultivar types as explanatory factors that significantly influenced disease presence/absence on farm. Access to training had a negative and significant (*P* = 0.009) influence on XW presence on farm, suggesting that the 8% of the farms without XW were also those that had received training on XW management. In contrast, 92% of the farms with XW had a high likelihood of having the susceptible ABB banana cultivar types (*P* = 0.029) (**Table 2**). A linear regression model for XW plant incidence on-farm selected the number of banana cultivars, as the key factor influencing XW incidence on a farm (**Table 2**). The number of cultivars on farm and the application of SDSR, one of the recommended control practices led to a decline in XW incidence, while incidence was increased on farm by the presence of the susceptible ABB banana types. However, only the number of cultivars had a significant effect at *P* = 0.05. The logistic regression and the linear regression models, respectively, explained 29.3% (null deviance: 2.7569 on 36 degrees of freedom (d.f.) and residual deviance: 1.9503 on 34 d.f.) and 18.1% (model *p*-value of 0.083) of the variation in XW presence/absence and incidence on farm, suggesting likely landscape effects not explored in this study played an important role in XW incidence on farm.

**Table 2:** Model outcomes of regressions of Xanthomonas wilt (XW) presence (as binary scores) and Xanthomonas wilt incidence on farm as response variables with different explanatory variables.

	Parameter estimate	Standard error	*t*-value	Pr (>| t| )
**Xanthomonas presence on farm**				
Intercept	0.62	0.17	3.53	0.0012^**^
Presence of ABB banana types	0.40	0.17	2.29	0.0286^*^
Household access to training	-0.23	0.08	-2.76	0.0094^**^
**Xanthomonas wilt incidence on farm**				
Intercept	21.17	17.81	1.19	0.2431
Number of banana cultivars on-farm	-4.62	2.25	-2.06	0.0478^*^
Presence of ABB banana types	26.01	16.83	1.55	0.1318
Application of SDSR	-13.48	8.12	-1.66	0.1064


*SDSR denotes ‘single diseased stem removal.’ Significance codes: 0.001 ‘^∗∗^’ 0.01 ‘^∗^’ 0.05.*

### Synthesis of the Risk Posed by Potential Alternative Host Plants

**Table 3** summarizes the different risk criteria and how they ranked across the crops in this study based on laboratory, screenhouse and field observations; and expert knowledge. In the screenhouse pot trials only *Canna* sp. was found to be highly susceptible whereas other crops were resistant (**Table 3**). The risk to/from *Canna* sp. and sugarcane could be increased by their perennial nature and propagation through the rhizome. Overall, the risk from *Canna* spp. could be ranked as moderate-high while low-moderate for sugarcane and none-very low for the other crop species in this study.

**Table 3:** Ranking of the risk of the different crops/weeds to perpetuate Xanthomonas wilt disease on banana plants on farms.

Criteria	*Canna* sp.	Wild sorghum	Millet	Sorghum	Taro	Maize	Sugarcane
Susceptibility to Xcm (based on ability to induce symptoms)	5	0	2	2	0	0	0
Recovery of Xcm from tissues	5	2	2	1	1	1	2
Life span	5 (Perennial – high risk of sustaining infection)	0 (annual)	0 (Annual no risk)	0 (Annual)	0 (Perennial but not a suitable host)	0 (Annual not a suitable host)	5 (Perennial)
Mode of propagation	5 (Dispersed through the rhizome, thus able to pass infection to plantlets)	0 (Seed dispersal)	0 (Seed dispersal)	0 (Seed dispersal)	0 (Not a suitable host)	0 (Seed dispersal)	4 (Dispersed through the rhizome and cuttings. Can potentially pass infection to plantlets)
Risk of spread though tools	5	1	1	1	0	1	3
Presence in banana fields (Likely to vary from place to place)	3 (52% of farms in Uganda)	2	1	2	3	3	3
Field assessment	1 (Only isolated from 0.02% of sampled plants)	Not assessed	Not assessed	Not assessed	Not assessed	Not assessed	Not assessed
Over all risk rating	Moderate-high	None-very low	None-very low	None-very low	None	None-very low	Low-moderate


*For the different criteria, rank 0 denotes no risk while 5 denotes a high risk.*

## Discussion

Alternative host plants play an important role in the spread and perpetuation of several plant pests and diseases. Several crop and weed species have been suspected to potentially play a role in the spread or survival of the XW disease-causing pathogen, Xcm.

In the current study, Xcm caused symptoms similar to those in banana in 96% of *Canna* sp. plants. This confirms the findings of Ssekiwoko et al. (2006a) and Chala et al. (2016), who observed symptoms in *Canna* spp. after inoculation with Xcm. The longer incubation period in *Canna* sp. plants could be attributed to the fact that fully grown *Canna* sp. plants were used in contrast to the other plants species. In the current study, Xcm was successfully re-isolated from 47% of the *Canna* sp. plants in the screenhouse study and confirmed with Xcm specific primers, a component not present in previous studies. Given the high symptom incidence in *Canna* sp. (96%), the observed level of Xcm recovery/re-isolation (47%) in *Canna* sp. was relatively low, a fact that could be attributed to the growth of saprophytes in the severely affected and decomposing plants which were assessed at later stages of sampling. Isolation of Xcm from banana plants with advanced disease symptoms has also been reported to be low due to the onset of decomposition and competition from saprophytes (Mwebaze et al., 2006; Were et al., 2015). Koch’s postulates showed, however, that the isolates from *Canna* sp. could induce disease in banana plantlets. These lab and screenhouse findings coupled with the high prevalence of this weed on banana farms in east and central Africa suggests that *Canna* spp. could pose a risk to the management of the XW disease.

Yet, in the field assessment only 0.02% of the field-grown *Canna* plants had Xcm. Moreover, presence/absence and incidence of XW on the studied farms was not influenced by the presence of *Canna* spp. on farm. A close examination of the *Canna* spp. plants in the field revealed that they have persistent floral bracts. In banana, cultivars with semi or persistent male/neuter flowers have been reported to escape insect-mediated infections (Tripathi and Tripathi, 2009; Blomme et al., 2014). Thus, the risk of *Canna* spp. on farm is likely to be limited to tool transmission mostly during weeding. The use of farm tools has been reported to potentially spread the Xcm bacteria from an infected banana plant to disease-free plants within or across fields (Ocimati et al., 2013). The weeding/land preparation periods (including banana leaf and sucker trimming) have been reported to be often followed by higher incidences of XW disease (Mgenzi Byabachwezi, 2016, personal communication). *Canna* spp. can propagate through the underground rhizome and as such Xcm infections can spread from one plant to other attached plants leading to disease persistence on a farm. In the current study, the attached suckers and sprouts in some of the symptomatic or dead *Canna* sp. plants, for example, were observed to succumb to the inoculation of the parent plants whereas in other pots the attached lateral shoots did not show disease symptoms even after death of parent plants. Based on the above findings we therefore rate the risk of *Canna* spp. to perpetuate XW on-farm as moderate to high.

Among the cereals inoculated with Xcm, symptoms were only observed in millet and sorghum plants, though the plants eventually recovered. The recovery suggests that the resistance mechanisms of these species could overcome the pathogen. Pathogen virulence is often directly correlated with pathogen replication, with higher levels of replication resulting in increased damage to the host (Ebert and Bull, 2008). However, virulence and pathogen replication can be decoupled (Margolis and Levin, 2008), especially if the host mounts an appropriate immune response against novel pathogens (Graham et al., 2005). Chala et al. (2016) also reported typical XW symptoms after Xcm inoculations into sorghum and millet. The appearance of symptoms in cereals and the taxonomic similarity of Xcm to *X. vasicola*, a pathogen of sorghum, maize and sugarcane should be a source of concern as this suggests a potential risk of a jump from one host to another. We, however, rate the risk of Xcm to these crops and from these crops to banana to be low mainly due to (i) the short annual cycle of these crops and the inability of Xcm to survive in absence of the host, (ii) the lack of infection arising from insects, and (iii) their dispersal through seeds and other criteria in **Table 3**. These cereals, unlike banana that is exposed to XW spread through insects foraging for nectar and pollen (a key mode of XW spread) are wind and/or self-pollinated. No symptoms were observed in maize and sugarcane. The absence of symptoms in maize and sugarcane is in agreement with earlier findings by Yirgou and Bradbury (1974) or Ashagari (1985) but contrasts findings from Karamura et al. (2015) and Chala et al. (2016) who reported typical Xanthomonas symptoms in sugarcane and maize, respectively, following inoculation with Xcm. No symptoms were also observed in wild sorghum contrary to reports by Chala et al. (2016). Taro a common banana intercrop reported to suffer from wilts by farmers in XW landscapes did not succumb to Xcm inoculation, suggesting that it is resistant.

Differences in results between the above studies could potentially arise from the differences in the varieties used and differences in virulence of Xcm isolates. Karamura et al. (2015) used six different isolates from different sites in the ECA region, while Chala et al. (2016) used three isolates arising from banana, wild enset and domesticated enset. Chala et al. (2016) reports different levels of susceptibility of banana, enset and the potential alternative hosts to these three isolates. This is strengthened by the fact that four isolates recovered from millet, wild sorghum (2 isolates) and sugarcane in this study had different levels of virulence, with a higher virulence observed in one of the isolates from wild sorghum, followed by that in millet and the least in the second wild sorghum isolate (cf. **Figure 8**). Evaluating a wide range of isolates from different geographical setups would therefore be vital. We rate the risk of wild sorghum and maize to be very low due to their short annual cycle and mode of pollination (i.e., wind pollinated) that prevents insect-mediated infections (**Table 3**). Sugarcane like other cereals are wind pollinated but are perennial and reproduce through cuttings and the underground rhizomes. Thus, in case Xcm gets adapted to it, it could be of importance in perpetuation of the problem of XW disease in banana. With tool-mediated XW spread and the observed increase in cases of banana–sugarcane intercrops (e.g., **Figure 9A**), sugarcane could potentially gain importance as an alternative host to Xcm. Based on the screenhouse and laboratory results, the characteristics of the sugarcane plant and its management, would propose a low –moderate risk score from/for sugarcane (**Table 3**).

Xcm was isolated from all the plant species after artificial inoculation with an Xcm isolate from banana in this study, irrespective of presence or absence of symptoms. Xcm re-isolation has also been reported for maize (Aritua et al., 2008; Karamura et al., 2015) and sugarcane (Karamura et al., 2015). In this study, isolates from maize and taro did not cause disease in banana plantlets. Rutikanga et al. (2016) also reported the isolation of Xcm from maize, beans and sweet potato plant parts and soils around the stems of these plants. The isolates though confirmed to be Xcm with PCR did not cause disease in banana plantlets (Rutikanga et al., 2016). The multiplication of bacterial pathogens *in planta* irrespective of the plant being a non-host or a host has been reported (Canteros et al., 1991; Hodson et al., 1995; Vidhyasekaran, 2002). The bacteria in non-hosts have been reported to, however, remain static or to decline after some time (Canteros et al., 1991; Vidhyasekaran, 2002). Novel host-pathogen interactions have not been under direct selection (Antonovics et al., 2013), and hence the degree of pathogen virulence is likely to be maladaptive for both the novel host and the pathogen. Host phylogeny has been considered a key factor in determining the susceptibility of novel hosts (Gilbert and Webb, 2007; de Vienne et al., 2009) because species closely related to the natural host of a pathogen tend to be more susceptible since the pathogen develops specialized adaptations to its natural host such as binding to host receptors, avoiding immune responses or utilizing host resources, and these break down if the environment provided by the novel host is too different (Longdon et al., 2014). We postulate that the failure of Xcm isolates from non-host plants to cause XW symptoms in banana may be due to loss or modification of key genes involved in virulence in the pathogenicity island of the Xcm genome. The loss of such loci could be attributed to the variation in fingerprint patterns compared to the original isolate (control). The ability of Xcm re-isolations from sugarcane and wild sorghum to cause disease in banana even though they did not show any symptoms in sugarcane or wild sorghum raises a big concern for the management of the disease as several plant species have been reported to act as symptomless hosts to pathogens. Age of plants (time of re-isolation) did not affect the recovery of the bacteria from the inoculated plants.

The observation of symptoms in some of the non-host plants in this study and earlier studies could be a cause for concern as several plant pathogens have been reported to evolve by host jumps followed by specialization (Grünwald and Flier, 2005; Raffaele et al., 2010). Species in the Phytophthora clade 1c are reported to have evolved through host jumps and subsequent adaptive specialization on plants from four dissimilar botanical families (Grünwald and Flier, 2005). In the group of xanthomonads, Coutinho et al. (2015) reported a significant host jump of *Xanthomonas vasicola* from sugarcane to eucalyptus. It is also not known for how long such pathogens need to survive in the non-host plants to warrant concern for management, especially given the reported closeness of Xcm to pathogens such as *X. vasicola* that are pathogens to some of these non-host cereal plants. For example, Peixoto et al. (2007) isolated *Xanthomonas campestris* pv. *viticola* from non-host plants such as *Alternanthera tenella, Amaranthus* sp., and *Glycine max*. These plants and grapevine (host) developed typical symptoms of bacterial canker when they were inoculated with the recovered isolates. However, the plant/crop spp. in this study were not reinoculated with isolates recovered from them.

Xanthomonas wilt presence and incidence on farm increased with the ABB banana types on farm. The ABB types, in addition to attracting many insect vectors, have floral bracts and neuter flowers that readily fall off leaving open fresh wounds on the rachis/flower stalk that are easily colonized by bacteria accidentally deposited by foraging insects (Blomme et al., 2014, 2017a). XW incidence also declined with increasing number of banana cultivars on farm. Crop or cultivar mixtures have been reported to impede disease and pest spread and damage (Poeydebat et al., 2016; Vidal et al., 2017; Li et al., 2018). High diversity of banana cultivars has been reported to suppress banana weevils, nematodes and black sigatoka in Uganda (Mulumba et al., 2012). The effect of mixtures on diseases can be through a dilution effect/decrease in the susceptible host, change in microclimate, pathogen dispersal rate and vector population and behavior, host alteration, and pathogen inhibition (Boudreau, 2013; Vidal et al., 2017). This study for the first time suggests a possible role of cultivar mixtures in reducing or slowing XW spread on farm. This effect of banana cultivars can be attributed to the effect of dilution on the susceptible ABB types and a reduced insect vector access to susceptible cultivars. XW incidence on farm was also influenced by singly removing diseased stems/plants on farm, i.e., SDSR application. SDSR is one of the recommended practices for managing XW disease on farms. SDSR has been reported to drastically reduce XW incidence and lead to recovery of infected banana fields if regularly and consistently applied (Blomme et al., 2017b). No associations were observed between XW presence/incidence with crop mixtures and agroforestry practices, possibly due to the homogeneity of the farms in the study region. The models only explained between 18 and 29% of the observed variation on-farm. This could be attributed to the fact that other factors responsible for disease spread on farm such as use of contaminated tools and un-certified seed were homogenous across farms. Landscape level factors could have also possibly dominated the studied factors within the individual farms. At landscape level, XW spread, incidence and severity has been reported to be influenced by altitude, temperature, precipitation, trade in diseased fresh products and the level of collective application of disease control measures among others.

## Conclusion

In the current study, despite a low recovery (0.02%) of Xcm from field-based *Canna* spp. plants, the high susceptibility of *Canna* sp. to Xcm in the screenhouse coupled to the pathogenicity of the Xcm isolates recovered from these plants to banana makes them suitable alternative hosts to Xcm. More still, given the perennial nature of *Canna* spp. and its propagation through the rhizome, even at low incidences, *Canna* spp. could act as a reservoir of Xcm and perpetuate XW disease, leading to a moderate-high risk from this weed. Isolates recovered from sugarcane and wild sorghum (another important weed species) also caused disease in banana though these plants were resistant following inoculation with Xcm. Given sugarcane and some species of wild sorghum, e.g., Johnson grass [*Sorghum halepense* (L.) Pers] are perennial and reproduce through the rhizome, they could potentially become important alternative hosts if the bacteria gets adapted to them. Efforts for managing XW will thus need to be broadened to management of some of the alternative host plants, especially *Canna* spp. In contrast, the risk from the grain cereals is deemed as none to very low, given the grain cereals were resistant, are annual in nature and propagate through seed. More still, XW cannot survive in decomposing or dry host tissues. Efforts to diversify agroecosystems therefore need to consider the interaction of pathogens of given crops with other plants in the system. On farmers’ fields, XW presence and incidence had no association with the potential alternative hosts, especially *Canna* spp. but rather with the presence of susceptible ABB *Musa* genotypes, number of cultivars on farm and access to training on XW management. The models accounted for only between 18–29% of the variation in XW presence on farm, suggesting a possible role of other factors on farm. There is also need for in-depth study of the genomes of the Xcm isolates retrieved from the potential alternative host plants.

## Author Contributions

WO conceived and developed the research concept, conducted the experiments, collected and analyzed the data, and wrote the manuscript. EW shaped the research concept, supported the experimentation, and collected and wrote the data. JG and PT guided and supervised the experiments and supported the writing of the manuscript. GN shaped the research concept and supported the establishment of experiments. GB shaped the research concept, supervised the study, and supported the writing and editing of the manuscript.

## Conflict of Interest Statement

The authors declare that the research was conducted in the absence of any commercial or financial relationships that could be construed as a potential conflict of interest. The reviewer DS declared a past co-authorship with several of the authors WO, GN, and GB to the handling Editor.

## Footnotes

1 https://www.ncbi.nlm.nih.gov/tools/primer-blast/primertool.cgi?ctg_time=1532089551&job_key=wsgdcVNWXv55wFvFVqV_9yy-bsUBrXXYAA&CheckStatus=Check
